# Non-organ-specific autoantibodies with unspecific patterns are a frequent para-infectious feature of chronic hepatitis D

**DOI:** 10.3389/fmed.2023.1169096

**Published:** 2023-06-14

**Authors:** Lennart Hermanussen, Sibylle Lampalzer, Jan-Hendrik Bockmann, Annerose E. Ziegler, Felix Piecha, Maura Dandri, Sven Pischke, Friedrich Haag, Ansgar W. Lohse, Marc Lütgehetmann, Christina Weiler-Normann, Julian Schulze zur Wiesch

**Affiliations:** ^1^Department of Medicine (Gastroenterology, Hepatology, Infectious diseases, and Tropical Medicine), University Medical Center Hamburg-Eppendorf (UKE), Hamburg, Germany; ^2^German Center for Infection Research (DZIF), Hamburg-Lübeck-Borstel-Riems Site, Hamburg, Germany; ^3^Institute of Immunology, University Medical Center Hamburg-Eppendorf, Hamburg, Germany; ^4^Institute of Medical Microbiology, Virology and Hygiene, University Medical Center Hamburg-Eppendorf (UKE), Hamburg, Germany; ^5^Department of Medicine and Martin Zeitz Centre for Rare Diseases, University Medical Centre Hamburg-Eppendorf, Hamburg, Germany

**Keywords:** hepatitis D virus, hepatitis B virus, autoimmune hepatitis, autoantibodies, viral hepatitis

## Abstract

Infections with hepatotropic viruses are associated with various immune phenomena. Hepatitis D virus (HDV) causes the most severe form of viral hepatitis. However, few recent data are available on non-disease-specific and non-organ-specific antibody (NOSA) titers and immunoglobulin G (IgG) levels in chronic hepatitis D (CHD) patients. Here, we examined the NOSA titers and IgG levels of 40 patients with CHD and different disease courses and compared them to 70 patients with chronic hepatitis B (CHB) infection. 43% of CHD patients had previously undergone treatment with pegylated interferon-α (IFN-α). The antibody display of 46 untreated patients diagnosed with autoimmune hepatitis (AIH) was used as a reference. The frequency of elevated NOSA titers (CHD 69% vs. CHB 43%, *p* < 0.01), and the median IgG levels (CHD 16.9 g/L vs. CHB 12.7 g/L, *p* < 0.01) were significantly higher in CHD patients than in patients with CHB, and highest in patients with AIH (96%, 19.5 g/L). Also, the antinuclear antibody pattern was homogeneous in many patients with AIH and unspecific in patients with viral hepatitis. Additionally, f-actin autoantibodies were only detectable in patients with AIH (39% of SMA). In CHD patients, IgG levels correlated with higher HDV viral loads, transaminases, and liver stiffness values. IgG levels and NOSA were similar in CHD patients irrespective of a previous IFN-α treatment. In summary, autoantibodies with an unspecific pattern are frequently detected in CHD patients with unclear clinical relevance.

## 1. Introduction

Hepatitis D virus (HDV) infection is the most severe form of viral hepatitis. HDV is a defective RNA virus and requires the hepatitis B surface antigen (HBsAg) of the hepatitis B virus (HBV) to complete its lifecycle. Worldwide, 15–20 million people suffer from a chronic HDV infection, which amounts to about 6–8% of the patients with a chronic HBV infection (CHB) (1). Chronic HDV/HBV co- or super-infection (CHD) is associated with higher rates of liver cirrhosis, hepatocellular carcinoma, and higher liver-related mortality compared to patients with HBV mono-infection (2–5). Chronic HDV infection has been a challenge in clinical hepatology in terms of diagnostics, monitoring, and therapy. For example, anti-HDV Immunoglobulin M (IgM) testing was used to diagnose ongoing hepatitis delta replication for a long time before HDV-RNA assays became available, and there have been incremental advances in the standardization of these molecular assays (6, 7). Antiviral treatment with pegylated Interferon-α (IFN-α) has been introduced around 10 years ago with low treatment response rates (8), and only lately therapy with bulevirtide received conditional approval in the EU (9).

Autoimmune hepatitis (AIH) is an immune-mediated chronic liver disease leading to (necro)inflammation, liver cirrhosis with all of its complications. AIH is characterized by elevated serum transaminases and immunoglobulin G (IgG) levels, detectable autoantibodies, and characteristic histopathological findings. Based on different autoantibody profiles, AIH can be classified into two subtypes, autoimmune hepatitis type 1 (AIH-1) and type 2 (AIH-2). (10, 11) Over the years there have been attempts to standardize the assessment of autoantibody profiles (12).

Notably, patients with chronic viral hepatitis may also develop high autoantibody titers (13, 14). Furthermore, overt extra-hepatic manifestations, such as vasculitis, polyarthritis nodosa, glomerulonephritis, dermatitis, polyarthralgia, arthritis, lung disease, and aplastic anemia may also occur (13, 15). Molecular mimicry and bystander activation have been proposed as possible mechanisms to explain the breakdown of self-tolerance caused by viral infection (16). Differentiating autoimmune phenomena in viral hepatitis from true autoimmune (liver) disease is of utmost clinical relevance: a concomitantly existing autoimmune liver disease would necessitate immunosuppressive treatment, which bears the risk of dampening control of the virus-induced liver disease.

Most of the antibodies in viral hepatitis are non-disease-specific and non-organ-specific antibodies (NOSA). Antinuclear antibodies (ANAs) were the first autoantibodies to be associated with AIH (17). Since NOSA have also been detected in patients with viral hepatitis, drug-induced liver injury, Wilson’s disease, alcohol-induced liver disease, non-alcoholic fatty liver disease, and a variety of extrahepatic autoimmune diseases, the specificity of ANAs for a specific (liver) disease is generally low (18).

Anti-smooth muscle antibodies (SMAs) are detected in up to 85% of patients with AIH-1 (19). The SMA titer is also of clinical significance since higher titers have higher AIH-specificity (11). Moreover, sub-specificity toward f-actin has been clearly associated with AIH (11). However, SMA can also be observed in up to 25% of patients with CHB, and chronic hepatitis C virus (HCV) infection (19–22).

The anti-liver-kidney microsomal type 1 antibodies (LKM1), which are targeted against cytochrome P450 CYP2D6 (23) are a hallmark in the diagnosis of AIH-2. In 1983, LKM autoantibodies were also described in CHD patients and later termed LKM-3 (24). These autoantibodies were present in 13–14% of Italian HDV carriers. The major LKM-3 autoantigen was identified as an epitope on family 1 UGTs (UGTl) (25).

Anti-mitochondrial antibodies (AMA) are typically present in patients suffering from primary biliary cholangitis (18) but have also been detected in a small number (3%) of patients with HCV (26). Anti-soluble liver antigen/liver-pancreas antibodies (SLA/LP) are the most specific for AIH-1 among all AIH-related antibodies. However, only a small proportion of patients with AIH show these antibodies that are associated with persistent disease (18, 27, 28).

In patients with CHD, basal cell layer (BCLA) and antithymic antibodies were frequently found, too (29).

The first evidence for autoantibodies in CHD was established as early as 1980 (30). However, in comparison to HBV and HCV infection (31, 32), data for autoimmune phenomena in chronic HDV infection is scarce (31). At the time when the last results on this topic were published nearly 30 years ago, antibody assessment was less standardized, and neither HDV PCR diagnostics, transient liver elastography, nor HDV interferon treatment was readily available (6, 7, 9).

A better knowledge of the autoantibody profiles of CHD might contribute to the understanding of immune-mediated disease progression and extrahepatic manifestations. Autoantibodies could also serve as suitable biomarkers to support the assessment of disease activity, to predict the clinical course, and treatment outcome.

In this study, we cross-sectionally evaluated the autoantibody titers and IgG levels in a single-center cohort of patients with CHD and different disease statuses and compared those to patients with CHB mono-infection and AIH. NOSA titers and IgG levels were correlated to HDV viremia, transaminases, and liver stiffness values from transient elastography as possible markers for the clinical status.

## 2. Materials and methods

### 2.1. Study population

We retrospectively identified and analyzed three different cohorts consisting of 46 patients with AIH, 42 patients with CHD, and 70 patients with CHB. Autoantibody titers were available in 46/46 patients of the AIH cohort, in 40/42 patients of the CHD cohort, and 69/70 patients of the HBV cohort. IgG levels were available in 44/46 patients of the AIH cohort, in 36/42 patients of the CHD cohort, and 61/70 patients of the HBV cohort.

All patients were treated in specialized outpatient clinics at the University Medical Center Hamburg-Eppendorf. The data were collected as part of the clinical routine visits between 2010 and 2019 and retrospectively analyzed. The diagnosis of AIH was secured by serological and histopathological findings as well as treatment response according to the EASL clinical practice guidelines (33). Viral hepatitis was excluded in patients of the AIH group. Inclusion criteria for participants in the CHD cohort were the confirmation of positive HBsAg status as well as proof of anti-HDV, not necessarily an existing viremia. In this present study, 19/41 (46%) of patients with HDV infection had a negative HDV PCR at the time of inclusion.

Patients receiving INF-α therapy within the last 6 months before inclusion, with a history of malignancy, HCV infection, or HIV were excluded. The study was approved by the local ethics committee (WF-035/17).

### 2.2. Antibody diagnostics

Testing for autoantibodies was performed by trained personnel at the Institute of Immunology at the University Medical Center Hamburg-Eppendorf. ANA, SMA, LKM-1, and AMA were assessed by immunofluorescence testing. HEp-2 Cells (human epithelioma cells) and tissue slides (Euroimmun, Lübeck, Germany) were incubated in accordance with the manufacturer’s instructions, and interpretation was performed manually using the Eurostar microscope (Euroimmun, Lübeck Germany). SLA was tested by ELISA using enzyme immunoassays from Euroimmun, Germany (11, 34). According to our internal laboratory criteria, we regarded ANA, SMA, and LKM-1 as positive at a titer of >1:80, AMA as positive at titers of ≥1:40, and SLA/LP as positive if ≥20 U/mL. IgG levels were deemed elevated above the cut-off 16 g/L.

### 2.3. Statistics

All data were collected in a Microsoft® Excel® (Version 12.3.6 for Mac) file and imported into IBM *SPSS* (Version 24.0 for Mac, Armonk, NY, United States, 2016) for statistical analysis.

Data are presented as absolute numbers and percentages or as the median and interquartile range (IQR). Nominal variables were compared using the Chi-Square test. Metric variables were analyzed using the Mann–Whitney-U-test because there was no normal distribution. Spearman’s rho was used for correlation analysis: the statistical significance is indicated following the correlation coefficient (*r*) (*p < 0.05, **p < 0.01).

## 3. Results

### 3.1. Baseline characteristics of the study population

The baseline characteristics of the three patient cohorts are summarized in Table 1. The three cohorts differed in terms of age: patients in the CHB cohort had a median age of 37 years, in the CHD cohort the median age was 45 years, and 54 years in the AIH cohort. Not surprisingly, there were significantly more men in the HDV cohort than in the AIH cohort since AIH is known to be a disease with predominance in women (60% versus 33%) (10).

**Table 1:** Baseline characteristics of the study population.

	AIH (*n* = 46)	CHD (*n* = 42)	CHB (*n* = 70)
Age (years)	54 (37–71)	45 (37–54)	37 (30–47)
Sex (female: male)	31:15	17:25	40:30
ASAT (U/l)	202 (68–801)	40 (26–92)	22 (18–31)
ALAT (U/l)	278 (76–758)	60 (32–117)	31 (19–47)
Viral load (U/ml) in patients currently treated with NAs	–	*n* = 7 HDV: 0 (0–12,200) HBV: 0 (0–200)	*n* = 11 0 (0–456)
Viral load (U/ml) in patients with a history of IFN-α treatment	–	*n* = 17 HDV: 32000 (870–220,000) HBV: 70 (6–790)	*n* = 58 2,400 (422–20,250)
Viral load (U/ml) in therapy naïve patients	–	*n* = 17 HDV: 0 (0–300) HBV: 378 (35–3,490)	*n* = 1 130
Transient elastography (kPA)	*n* = 27 8.2 (6.1–17.9)	*n* = 36 7.7 (6.2–14.0)	*n* = 40 5.3 (4.3–6.5)

Values are medians with an interquartile range (IQR).

In the CHB cohort, 16% were under treatment with nucleoside/nucleotide analogs (NA) and had a median viral load under the limit of detection (0–456 IU/mL); 1% had previously undergone treatment with IFN-α and had an HBV DNA load of 130 IU/mL; 83% were therapy naïve and had a median viral load of 2,400 (422–20,250) IU/ml.

In the CHD cohort, 17% of patients were under treatment with nucleoside/nucleotide analogs (NA) and had median HDV and HBV viremia underneath the detection limit (HDV RNA: 0–12,200 IU/mL; HBV DNA: 0–200 IU/mL); 43% of HDV patients had previously undergone treatment with IFN-α and had a median HDV viremia of 32,000 IU/mL (870–220,000) and a median HBV viremia of 70 (6–790) IU/ml; 40% were therapy naïve and had a median HDV viremia under the limit of detection (0–300 IU/mL) and a median HBV DNA load of 378 (35–3,490) IU/ml.

82% of AIH patients were treatment naïve at the time of study inclusion. 7% of patients had received steroids (prednisolone or budesonide) before, and another 11% had previously been treated with steroids and azathioprine.

### 3.2. IgG levels in patients with viral hepatitis compared to AIH patients

**Figure 1:**
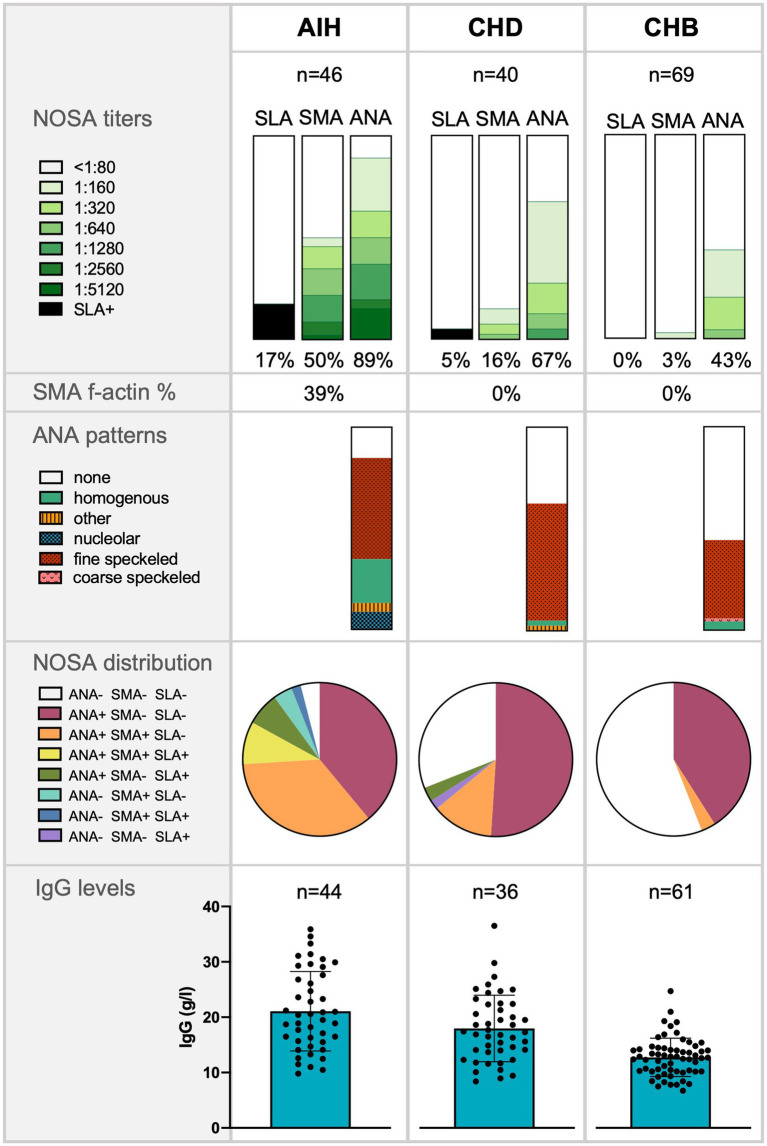
Frequency of non-disease-specific and non-organ-specific antibodies (NOSA), antinuclear antibodies (ANA) patterns, NOSA distribution, and Immunoglobulin G (IgG) levels in patients with autoimmune hepatitis (AIH), chronic hepatitis D (CHD), and chronic hepatitis B (CHB). The significance of IgG levels was tested with a Mann–Whitney-U test: AIH compared to CHD (*p* < 0.05*); CHD compared to CHB (*p* < 0.01**).

Patients with CHD had statistically higher IgG levels than patients with CHB (*p* < 0.01), but lower IgG levels than patients with AIH (*p* < 0.05). In the AIH cohort, the median IgG level was 19.5 g/L (15.5–27.4) and was elevated in 73% of cases. In the CHD cohort, IgG levels were measured with a median value of 16.9 g/L (12.3–22.4). Elevated IgG levels were detected in 54% of CHD patients. In the CHB cohort, the median IgG level was 12.7 g/L (10.2–14.3) and was elevated in 12% (Figure 1). In addition, patients with CHD showed a weak correlation between IgG levels and HDV viral load levels (*r* = 0.39*), whereas there was no correlation between IgG levels and HBV viral loads. Thus, patients with detectable HDV RNA (46%) had significantly higher IgG levels compared to patients with undetectable HDV RNA (*p* > 0.01). Patients with undetectable HDV RNA had similar IgG levels to patients of the CHB cohort. IgG levels were similar in viremic CHD patients regardless of the IFN-α treatment status. IgG levels also correlated weakly significantly with ANA titers in patients with CHD (*r* = 0.42*). In patients with AIH, IgG levels correlated weakly with SMA titers (*r* = 0.34*; Table 2).

**Table 2:** Spearman correlation analysis of ANA and SMA titers as well of IgG levels with laboratory parameters in all cohorts.

ANA titers with	AIH (*n* = 46)	CHD (*n* = 40)	CHB (*n* = 70)
ALAT	−0.144	0.118	−0.122
ASAT	−0.156	0.361*	−0.096
IgG level	0.071 (*n* = 44)	0.419 (*n* = 36)*	−0.087 (*n* = 61)
HBV viral load	–	−0.107	0.079
HDV viral load	–	−0.131	–
Age	0.285	−0.032	−0.093
Liver stiffness	−0.029	0.218	−0.037
SMA titers with			
ALAT	0.187	0.133	−0.041
ASAT	0.183	0.182	0.063
IgG level	0.342 (*n* = 44)*	0.098 (*n* = 36)	−0.067 (*n* = 61)
HBV viral load	–	−0.183	0.100
HDV viral load	–	0.165	–
Age	−0.202	−0.188	−0.085
Liver stiffness	0.002	0.526**	0.033
IgG level with	AIH (*n* = 44)	CHD (*n* = 36)	CHB (*n* = 61)
ALAT	0.217	0.510**	0.064
ASAT	0.330*	0.771**	−0.022
HBV viral load	–	−0.158	0.160
HDV viral load	–	0.389*	–
Age	−0.029	−0.209	−0.160
Liver stiffness	0.192	0.410*	0.364*

*p < 0.05, **p < 0.01.

### 3.3. Levels, staining patterns, and distribution of non-organ-specific antibodies in patients with viral hepatitis compared to AIH

Positive NOSA titers were found more frequently in the CHD cohort than in the CHB cohort (CHD 69% vs. CHB 43%, *p* < 0.01). However, positive NOSA titers were significantly less frequent in patients with HDV infection than in patients diagnosed with AIH (CHD 69% vs. AIH 96%, *p* < 0.01). Notably, the patterns differed between the cohorts: AIH patients displayed significantly more homogenous ANA autoantibodies than the other cohorts. In addition, AIH patients displayed significantly more f-actin autoantibodies than the other cohorts. Details of the ANA staining patterns are shown in Figure 1.

Positive ANA titers were less frequently detected in patients with CHD than in patients with AIH (CHD 67% vs. AIH 89%, *p* < 0.05). In addition, significantly fewer patients with HDV had high ANA titers of ≥1:320 than patients with AIH (CHD 28% vs. AIH 63%, *p* < 0.01). However, patients with HDV infection were more likely to have positive ANA titers than patients with CHB (CHD 67% vs. CHB 43%, p < 0.05). CHD patients with detectable HDV RNA were not more likely to have positive ANA titers or ANA titers of ≥1:320 compared to CHD patients without detectable HDV RNA.

In general, positive SMA titers were most frequently observed in patients with AIH (AIH 50% vs. CHD 16% vs. CHB 3%). Specifically, 91% of patients with positive SMA titers in the AIH cohort had high SMA titers of ≥1:320. Patients with CHD tended to have positive SMA titers more frequently than patients with CHB (CHD 16% vs. CHB 3%, *p* = 0.055). In addition, detectable HDV RNA tended to be associated with positive and high SMA titers ≥1:320 compared to patients with undetectable HDV RNA (>1:80: *p* = 0.08; ≥1:320: *p* = 0.08).

Elevated SLA titers were detected in one patient (3%) with CHD and eight patients (17%) diagnosed with AIH, but not in any patient with CHB.

Neither patients with viral hepatitis nor patients with AIH had positive AMA or LKM-1 titers.

Among CHD patients, ANA titers were higher in IFN-α naïve patients than in those who were treated with IFN-α at some time before (median 1:1280 vs. 1:160). SMA titers were detected independently of previous IFN-α treatment. Among the CHD patients who received IFN-α therapy 3/18 (17%) showed sustained response during follow-up. For these patients, NOSA titers were determined before and after treatment and remained unchanged.

The distribution of NOSA was analyzed for each cohort and visualized in Figure 1: Patients with CHB almost exclusively had elevated ANA titers with negative SMA and negative SLA titers. In patients with CHD and patients with AIH, positive ANA titers were also combined with positive SMA titers. However, this pattern occurred significantly less frequently in patients with CHD than in patients with AIH (CHD 13% vs. AIH 35%, *p* < 0.05). The CHD patient with positive SLA titer was negative for SMA and ANA.

NOSA titers did not correlate with HDV viral load levels.

### 3.4. Antibodies and clinical parameters

Transaminases, alanine-aminotransferase (ALAT) and aspartate- aminotransferase (ASAT) were significantly higher in the AIH cohort compared to the two viral hepatitis cohorts (*p* < 0.01). However, transaminases were significantly higher in patients with HDV infection compared to patients with CHB (*p* < 0.01). A moderate or weak correlation between transaminases (ALAT; ASAT) and IgG levels were observed in patients with CHD (*r* = 0.51**; *r* = 0.77**) and AIH (*r* = 0.22; *r* = 0.33*). Transaminase levels were significantly higher in patients with viremic HDV infection than in patients with undetectable HDV RNA, in whom transaminase levels did not exceed those of patients of the CHB cohort. Moreover, a weakly significant correlation between ANA titers and ASAT was seen in patients with HDV infection (*r* = 0.36*).

There was no statistical difference in liver stiffness values determined by transient elastography among the three different patient cohorts (in kPa: CHB 5.3 (4.3–6.5); CHD 7.7 (6.2–14.0); AIH 8.2 (6.1–17.9)). In CHD patients, liver stiffness values did not correlate with ANA titers (r = 0.22), but showed a moderate correlation with SMA titers (*r* = 0.53**). In the CHD cohort, liver stiffness values also correlated with HDV viral load (*r* = 0.52**) and IgG levels (*r* = 0.41*). CHD patients with liver cirrhosis (liver stiffness values >14 kPa) had significantly higher IgG levels compared to CHD patients with low liver stiffness values (< 6.5 kPa) (*p* < 0.05). Also, in CHB patients, IgG levels weakly correlated with liver stiffness values (*r* = 0.36*). In the AIH cohort, no correlation was found between liver stiffness values and ANA titers, SMA titers, or IgG levels.

### 3.5. Histopathological findings of all cohorts

Biopsies were available for 34 AIH patients, 12 patients with CHD, and 3 patients with CHB (Supplementary Tables 1–3). The median Desmet score for liver fibrosis/cirrhosis was similar for AIH and CHD patients (2/4). The biopsies of patients with CHB did not show fibrosis/cirrhosis (0/4). The median modified Ishak score (mHAI) was higher in AIH patients (9/18, IQR 4–16) compared to patients with CHD (6.5/18, IQR 2–10) and CHB (4,5/18, IQR 3–6), without reaching statistical significance.

## 4. Discussion

Infections with hepatotropic viruses have been associated with various immunopathological manifestations. An association between infection and autoimmunity is well documented, particularly for chronic infections with hepatitis B and hepatitis C viruses (31). In comparison, less data was available on the frequency and pattern of NOSA for CHD and in relation to the disease or treatment status (30, 35).

In chronically HDV-infected individuals, perforin-positive cytotoxic CD4+ T cells accumulate and have been implicated in contributing to the severity of HDV related liver infection (36–38). Also, HDV infection was shown to augment the antiviral state of the hepatocytes, chemokine production, and antigen presentation (39, 40). Though the liver damage that results from chronic HDV infection is considered to be primarily mediated by the immune system (37, 41), little is known about the role of autoantibodies.

In this current study, we cross-sectionally examined available NOSA titers and IgG levels in 42 patients with CHD and 116 controls consisting of 70 patients with CHB, and 46 patients diagnosed with AIH. Because patients with CHD were compared to patients with HBV mono-infection, differences in autoimmune phenomena, represented by autoantibody titers and IgG levels, could be associated with the addition of HDV infection.

This study is limited by its retrospective design, the absence of multicenter validation, and the small sample size. Also, the three cohorts were not matched for age, sex, or degree of liver damage. Only very selected autoantibodies were analyzed as used in the standard workup of elevated liver enzymes. This panel is primarily matched to the autoantibody profile of AIH, PSC, and PBC.

Here, almost exclusively patients with HDV genotype 1 infection were included, as this is the most common genotype circulating in Europe (42). However, recent studies have shown differences in spreading kinetics, treatment outcome, and disease courses between variant HDV genotypes (43, 44). Therefore, it would be of interest whether the autoantibody profile differs, too.

Elevated serum IgG levels are considered a characteristic feature of AIH, as they are detected in up to 85% of patients and are part of the diagnostic score in AIH (45). We found elevated IgG levels in 73% of patients with AIH, and only in 12% of patients with CHB, but also in 54% of patients with CHD. Hartl et al. previously suggested a correlation between ANA/SMA titers and IgG levels in patients with AIH, but IgG levels were not related to the degree of liver fibrosis or intrahepatic inflammatory activity (45). In this current study, there was also a significant correlation between IgG and ANA titers in CHD patients and SMA and IgG in AIH patients. However, there was also a significant correlation between IgG and transaminases, suggesting a relationship between IgG and intrahepatic inflammatory activity in CHD and AIH patients. Also, in the CHD cohort IgG levels and HDV viral loads correlated with higher liver stiffness values. Patients with HDV viremia had significantly higher IgG levels than patients without detectable viremia.

Most data available on the frequency of autoantibodies in CHD patients is historic, when the methodologies applied differend, were less standardized, and IFN-α treatment was not available. In this study, patients with CHD were significantly more likely to have positive/high NOSA titers than patients with CHB (CHD 69% vs. CHB 43%; *p* < 0.01), but less likely to have positive NOSA titers than patients with AIH (CHD 69% vs. AIH 96%; p < 0.01). The prevalence of positive NOSA titers in AIH patients determined in our study was consistent with the literature (46). In a study by McFarlane et al. from 1995, about 20% of CHD patients (*n* = 27) had positive NOSA titers, similar to the included patients with HBV mono-infection (*n* = 22) (35). In a study with 325 Chinese patients diagnosed with CHB, 58.2% showed positive NOSA titers at the same cut-off value as in our analysis, which was significantly higher than in healthy controls (6.7%) (47).

In line with our results, ANA were among the most frequent NOSA in the CHB patients of the Chinese cohort (47). The data base for ANA in CHD is scarce. In contrast to our results, in 1986, Zauli et al. found the prevalence of ANA to be significantly lower in CHD patients (9%) than in CHB patients (48).

Patients with AIH were significantly more likely to have positive SMA titers and, in particular, to have high titers (≥ 1:320) than patients with CHD or CHB. However, 8% of patients with CHD also showed SMA titers, while these antibodies were not present in CHB patients. While patients with HBV mainly had isolated elevated ANA titers, patients with HDV and AIH also had combined elevated ANA and SMA titers. In a previous study from 1986, the prevalence of SMA in CHD did not significantly differ from chronic hepatitis HBV mono-infection (48). Here, SMA titers correlated with liver stiffness values in CHD patients but not in the other cohorts. Larger studies should be conducted to validate whether high SMA titers are indeed associated with activity or prognosis of the HDV liver disease.

SLA is considered the most specific marker for AIH among all AIH-related antibodies. It is still controversial whether the detection of SLA is associated with severe courses of AIH (18, 27).

Rarely, do HCV/LKM-1 positive patients progress to LKM-1 positive autoimmune hepatitis (49). Positive LKM-1 was not detected in any patients of this current study. Whereas the presence of LKM-1 is a typical feature of AIH-2, only patients with AIH-1 were included in this study. In this study, some CHD patients had high mHAI scores. Whether HDV infection – as has been described for chronic HCV infection – can indeed trigger an AIH, is not fully elucidated.

On IFN-α therapy, HCV/LKM-1 positive patients experience increases in aminotransferase levels, occasionally of such magnitude to warrant suspension of treatment (50, 51). Subgroup analysis of CHD patients showed no differences in NOSA titers irrespective of a previous IFN-α-therapy. In the current study, CHD patients with elevated NOSA titers did not experience relevant increases in aminotransferase levels upon IFN-α treatment. In our study, 43% of the CHD patients underwent treatment with pegylated IFN-α, although only 17% of IFN-α treated HDV patients showed a sustained response (HH-CHD8, HH-CHD26, HH-CHD42, Supplementary Table 3). These patients showed similar NOSA titers before and after treatment. Interestingly, in another recently published case of CHD with clinical and histological stigmata of AIH, therapy with the HBV/HDV entry inhibitor bulevirtide led to rapid normalization of immunoglobulin levels (52). Further studies are needed to assess whether such a therapeutic approach may lead to a substantial reduction of immunoglobulin levels. The literature does not indicate benefits in treating CHD patients displaying high NOSA titers with immunomodulating agents such as steroids (53, 54).

The median mHAI score was the highest in the AIH cohort, though not significantly compared to the CHD and CHB cohorts. Interestingly, the CHD patient with the highest mHAI score (10/18) also had highly elevated ANA titers and IgG levels. However, by histology, it is not possible to distinguish between viral hepatitis and AIH with certainty, and elevated NOSA as well as IgG levels were detected in many patients of the CHD cohort. Of note, NOSA patterns differed significantly in patients with AIH and viral hepatitis: the pattern of ANA autoantibodies in AIH patients was significantly more often homogenous than in the other two patient groups. Also, AIH patients displayed f-actin autoantibodies significantly more often than patients with viral hepatitis. If validated in larger studies, such differences in the NOSA titers and pattern may even be used to establish novel biomarkers to delimit the few patients with covert AIH from the majority of patients that demonstrate NOSA rather as an unspecific para-infectious immune phenomenon.

Notably, high NOSA titers might predict the development of autoimmune disease even after sustained HDV control (55). Arbuckle et al. found that ANAs with a dilution titer of 1:120 were present in 78% of lupus erythematosus patients studied 3–9 years before clinical manifestation (56). One patient of the CHD cohort also had cryoglobulins and was diagnosed with membranoproliferative glomerulonephritis (HH-CHD41, Supplementary Table 3). Additionally, prospective studies examining the presence of high autoantibody titers in an HDV cohort are needed to assess their significance as early predictors of autoimmune disease or as a singular para-infectious phenomenon. In this context, it seems important to investigate the prevalence of HDV infection in other autoimmune diseases. Further studies should assess the prevalence of additional clinical symptoms (arthralgias, sicca symptom) and autoantibodies typical of other autoimmune diseases. For example, anti-SSA and anti-SSB in Sjögren’s syndrome, and cryoglobulins have been repeatedly found in patients with chronic hepatitis B, C, and E infection (34).

Possible mechanisms responsible for the immunopathology seen in chronic viral infections, in general, include molecular mimicry, impairment of regulatory T-cell activity, and polyclonal activation of B lymphocytes (31, 57, 58). Polyclonal activation of B cells seems to be a likely explanation of the correlation of IgG levels with HDV disease activity and severity. The persistence of elevated NOSA titers in non-viremic HDV patients could be due to the formation of persisting dysfunctional atypical memory B cells (61, 62). The role of B cells in CHD needs to be clarified in future studies.

In summary, autoantibodies are frequently detected as a para-infectious feature of CHD patients with unclear clinical significance.

## Data availability statement

The original contributions presented in the study are included in the article/Supplementary material, further inquiries can be directed to the corresponding author.

## Ethics statement

The studies involving human participants were reviewed and approved by Hamburger Ethikkammer. Written informed consent for participation was not required for this study in accordance with the national legislation and the institutional requirements.

## Author contributions

JSZW, ML, and CW-N initiated and supervised the study. LH, SL, and JSZW wrote the first draft of the manuscript. SL, LH, and CW-N performed analyses and generated data. FH, MD, AWL, AZ, FP, and J-HB discussed the data and corrected the manuscript. All authors contributed to the manuscript and approved the final submitted version.

## Funding

JSZW, AWL, MD, and ML are funded by the DZIF. JSZW is funded by DFG SFB1328, and the EU (Thervac B).

## Conflict of interest

The authors declare that the research was conducted in the absence of any commercial or financial relationships that could be construed as a potential conflict of interest.

## Publisher’s note

All claims expressed in this article are solely those of the authors and do not necessarily represent those of their affiliated organizations, or those of the publisher, the editors and the reviewers. Any product that may be evaluated in this article, or claim that may be made by its manufacturer, is not guaranteed or endorsed by the publisher.
