# Supplementary material for: Non-organ-specific autoantibodies with unspecific patterns are a frequent para-infectious feature of chronic hepatitis D
Source: Front Med (Lausanne). 2023 Jun 14;10:1169096. doi: 10.3389/fmed.2023.1169096 (PMC10300640; doi:10.3389/fmed.2023.1169096)
Supplement: Supplementary file 1 [file Table_1.DOCX]

| **No.** | **Sex** | **Age** | **Therapy** | **liver stiffness (kPa)** | **ASAT** | **ALAT** | **AP** | **GGT** | **IgG** | **ANA Titer** | **ANA Pattern** | **SMA** | **F-Actin** | **SLA** | **Biopsy** | **Fibrosis** | **mHAI** |
| --- | --- | --- | --- | --- | --- | --- | --- | --- | --- | --- | --- | --- | --- | --- | --- | --- | --- |
| **1** | **f** | **68** | **0** | **-** | **53** | **46** | **321** | **965** | **9,8** | **640** | **4** | **0** |  | **< 2** | **1** | **4** |  |
| **2** | **f** | **29** | **0** | **-** | **469** | **739** | **177** | **51** | **18,7** | **640** | **1** | **0** |  | **< 2** | **1** | **0** | **9** |
| **3** | **f** | **30** | **0** | **6,4** | **82** | **129** | **123** | **298** | **29,6** | **320** | **6** | **1280** |  | **< 2** | **1** | **2** | **4** |
| **4** | **m** | **79** | **prednisolone, azathioprine** | **-** | **199** | **264** | **250** | **1027** | **21** | **1280** | **6** | **1280** |  | **< 2** | **1** | **2** | **16** |
| **5** | **m** | **52** | **0** | **42,9** | **343** | **275** | **360** | **1142** | **16,5** | **320** | **2** | **0** |  | **7,32** | **1** | **4** | **9** |
| **6** | **f** | **58** | **0** | **6,3** | **44** | **77** | **84** | **41** | **10,5** | **640** | **7** | **0** |  | **< 2** | **1** | **2** | **7** |
| **7** | **f** | **76** | **prednisolone** | **4,9** | **176** | **365** | **92** | **179** | **12,5** | **1280** | **2** | **0** |  | **< 2** | **1** | **1** | **5** |
| **8** | **f** | **53** | **0** | **10,7** | **864** | **1432** | **284** | **733** | **18,9** | **1280** | **4** | **80** | **30,5** | **3,8** | **1** | **2** | **12** |
| **9** | **f** | **39** | **prednisolone** | **4,7** | **64** | **134** | **143** | **150** | **22,8** | **5120** | **8** | **320** |  | **3,2** | **1** | **2** | **16** |
| **10** | **m** | **47** | **prednisolone** | **5,9** | **276** | **801** | **85** | **154** | **13,3** | **1280** | **8** | **320** |  | **180** | **1** | **0** | **9** |
| **11** | **m** | **28** | **0** | **11,2** | **2491** | **2888** | **134** | **178** | **23,6** | **0** |  | **0** |  | **5** | **1** | **1** | **9** |
| **12** | **f** | **43** | **0** | **6,3** | **80** | **144** | **89** | **62** | **17,1** | **160** | **1** | **0** |  | **> 200** | **1** | **3** | **8** |
| **13** | **m** | **70** | **0** | **-** | **862** | **1751** | **164** | **291** | **11** | **160** | **1** | **640** |  | **10,2** | **1** | **0** | **9** |
| **14** | **f** | **30** | **0** | **7,4** | **435** | **463** | **138** | **32** | **18,2** | **0** |  | **0** |  | **3,8** | **1** | **0** | **6** |
| **15** | **f** | **48** | **0** | **-** | **1539** | **1544** | **162** | **126** | **21,2** | **160** | **8** | **1280** |  | **2,9** | **1** | **0** | **10** |
| **16** | **f** | **71** | **prednisolone, azathioprine** | **9,3** | **205** | **281** | **87** | **232** | **14,1** | **5120** | **3** | **640** | **90,7** | **4,4** | **1** | **0** |  |
| **17** | **f** | **51** | **0** | **10,4** | **57** | **74** | **80** | **91** | **27,6** | **640** | **1** | **2560** |  | **3,8** | **1** | **3** | **9** |
| **18** | **m** | **69** | **0** | **-** | **16** | **21** | **46** | **22** |  | **160** | **1** | **0** |  | **4,3** | **1** | **4** | **12** |
| **19** | **f** | **55** | **0** | **18,6** | **440** | **551** | **217** | **207** | **29,3** | **160** | **1** | **320** |  |  | **1** | **3** |  |
| **20** | **f** | **78** | **0** | **21,3** | **499** | **652** | **188** | **341** | **34,6** | **1280** | **4** | **1280** | **94,8** | **< 2** | **1** | **4** | **12** |
| **21** | **m** | **37** | **0** | **-** | **73** | **172** | **81** | **62** | **20,9** | **1280** | **5** | **0** |  | **> 200** | **1** | **1** | **8** |
| **22** | **f** | **76** | **0** | **-** | **53** | **54** | **102** | **71** | **26,1** | **5120** | **9** | **40** |  | **< 2** | **1** | **1** | **5** |
| **23** | **f** | **77** | **0** | **-** | **74** | **92** | **121** | **325** | **20,4** | **640** | **4** | **80** |  | **20,5** | **1** |  |  |
| **24** | **m** | **75** | **0** | **-** | **1249** | **724** | **203** | **195** | **35,9** | **160** | **2** | **0** |  | **< 2** | **1** | **3** | **9** |
| **25** | **f** | **61** | **azathioprine, budesonide** | **-** | **26** | **26** | **111** | **25** | **12,6** | **160** | **1** | **320** |  | **< 2** | **1** | **3** | **6** |
| **26** | **m** | **77** | **0** | **-** | **484** | **464** | **118** | **211** | **33,3** | **5120** | **3** | **0** |  | **< 2** | **1** | **4** | **16** |
| **27** | **m** | **30** | **0** | **-** | **480** | **584** | **110** | **185** | **31,1** | **320** | **1** | **2560** | **105** | **65,9** | **1** | **4** | **13** |
| **28** | **f** | **61** | **0** | **-** | **1216** | **744** | **219** | **533** | **11,5** | **160** | **1** | **160** |  | **< 2** | **1** | **4** | **11** |
| **29** | **f** | **50** | **0** | **6,1** | **1580** | **1309** | **198** | **548** | **31,4** | **1280** | **1** | **5120** |  | **< 2** | **1** |  |  |
| **30** | **m** | **74** | **0** | **-** | **969** | **1016** | **210** | **183** | **29,9** | **160** | **1** | **160** |  | **< 2** | **1** | **2** | **10** |
| **31** | **f** | **30** | **0** | **-** | **798** | **1668** | **87** | **135** | **18,9** | **160** | **3** | **1280** |  |  | **1** | **1** | **3** |
| **32** | **f** | **70** | **0** | **21,8** | **73** | **66** | **74** | **35** | **15,5** | **80** | **10** | **640** | **52,4** | **< 2** | **1** | **2** | **5** |
| **33** | **f** | **52** | **0** | **32,8** | **346** | **320** | **-** | **244** | **26,8** | **2560** | **11** | **2560** | **101** | **49,3** | **1** | **4** | **6** |
| **34** | **m** | **59** | **0** | **-** | **1415** | **1489** | **117** | **140** | **15,7** | **5120** | **3** | **0** |  | **4,4** | **1** | **2** | **8** |
| **35** | **f** | **25** | **prednisolone, azathioprine** | **7,1** | **20** | **20** | **64** | **28** | **14,7** | **2560** | **12** | **640** | **59,1** | **9** | **1** | **2** |  |
| **36** | **f** | **28** | **0** | **-** | **109** | **167** | **71** | **37** | **29,09** | **80** | **1** | **640** | **92,3** | **> 200** | **1** | **3** | **9** |
| **37** | **f** | **41** | **prednisolone, azathioprine** | **-** | **299** | **419** | **116** | **169** | **30,5** | **1280** | **1** | **1280** | **115** | **> 200** | **1** | **4** |  |
| **38** | **f** | **86** | **0** | **21,1** | **185** | **207** | **72** | **164** | **24,7** | **5120** | **8** | **0** |  | **2,3** |  |  |  |
| **39** | **f** | **71** | **0** | **-** | **95** | **155** | **79** | **34** | **14,12** | **320** | **1** | **0** |  | **2,5** | **1** | **0** | **9** |
| **40** | **f** | **51** | **0** | **-** | **69** | **60** | **118** | **20** | **17,7** | **5120** | **9** | **0** |  | **8,1** | **1** | **2** | **7** |
| **41** | **f** | **31** | **0** | **-** | **1664** | **2070** | **257** | **236** | **20,6** | **0** |  | **640** |  | **< 2** | **1** | **1** | **14** |
| **42** | **m** | **74** | **0** | **-** | **90** | **110** | **82** | **178** | **19,5** | **640** | **1** | **40** |  | **5,5** | **1** | **4** |  |
| **43** | **f** | **63** | **0** | **-** | **40** | **18** | **119** | **161** | **23,3** | **320** | **2** | **40** |  | **0** | **1** | **4** |  |
| **44** | **f** | **28** | **0** | **-** | **811** | **992** | **198** | **24** |  | **320** | **1** | **80** |  | **0** |  |  |  |
| **45** | **m** | **36** | **0** | **4,7** | **51** | **55** | **71** | **40** | **16,5** | **160** | **1** | **320** |  | **5,1** |  |  |  |
| **46** | **m** | **58** | **0** | **17,9** | **29** | **41** | **84** | **154** | **16,3** | **160** | **2** | **0** | **6** |  | **1** | **2** | **5** |
