# Supplementary material for: Non-organ-specific autoantibodies with unspecific patterns are a frequent para-infectious feature of chronic hepatitis D
Source: Front Med (Lausanne). 2023 Jun 14;10:1169096. doi: 10.3389/fmed.2023.1169096 (PMC10300640; doi:10.3389/fmed.2023.1169096)
Supplement: Supplementary file 2 [file Table_2.DOCX]

| **No** | **Sex** | **Age** | **Therapy** | **HDV Genotype** | **HDV viral load (IU/ml)** | **HBV Genotype** | **HBV viral load (IU/ml)** | **liver stiffness (kPa)** | **ASAT** | **ALAT** | **AP** | **GGT** | **IgG (g/l)** | **ANA Titer** | **ANA pattern** | **SMA** | **SLA** | **Cryo-globulins** | **Biopsy** | **Fibrosis** | **mHAI** |
| --- | --- | --- | --- | --- | --- | --- | --- | --- | --- | --- | --- | --- | --- | --- | --- | --- | --- | --- | --- | --- | --- |
| 1 | f | 54 |  |  | 0,00E+00 |  | 0,00E+00 | 6,8 | 32 | 32 | 75 | 12 | 8,38 | 80 | 1 | 0 | < 2 |  |  |  |  |
| 2 | f | 48 | INF 2004 |  | 0,00E+00 |  | 0,00E+00 | 7,3 | 26 | 32 | 94 | 15 | 8,93 | 80 |  | 0 | < 2 |  |  |  |  |
| 3 | m | 61 | INF 2012 |  | 6,00E+05 | D | 2,00E+03 | 14,4 | 42 | 84 | 60 | 100 | 9,45 | 0 |  | 40 | < 2 |  | 1 | 3 | 8 |
| 4 | m | 30 |  |  | 0,00E+00 |  | 7,00E+01 | 5,8 | 25 | 33 | 66 | 29 | 10,10 | 160 | 1 | 0 | < 2 |  |  |  |  |
| 5 | m | 45 |  |  | 1,00E+06 |  | 3,49E+03 | 10,1 | 44 | 95 | 69 | 39 | 10,5 | 0 |  | 80 | 88,6 |  |  |  |  |
| 6 | m | 33 |  |  | 0,00E+00 |  | 2,10E+04 | 12,6 | 22 | 19 | 110 | 48 | 11,6 | 160 | 1 | 80 | 4 |  |  |  |  |
| 7 | f | 49 |  |  | 0,00E+00 |  | 1,70E+03 | 5,2 | 13 | 15 | 54 | 14 | 11,7 | 80 |  | 0 | < 2 |  |  |  |  |
| 8 | m | 58 | INF 2013 | 1 | 0,00E+00 |  | 1,70E+04 | 6,1 | 26 | 28 | 73 | 19 | 11,8 | 160 | 2 | 0 | < 2 |  |  |  |  |
| 9 | m | 65 |  | 1 | 0,00E+00 |  | 3,50E+01 | 4,3 | 20 | 46 | 82 | 54 | 12,3 | 160 | 1 | 0 | < 2 |  | 1 | 2 |  |
| 10 | f | 42 |  |  | 0,00E+00 |  | 2,00E+06 |  | 30 | 30 | 105 | 17 | 12,3 | 0 |  | 40 |  |  | 1 | 0 | 2 |
| 11 | f | 45 | INF | 1 | 1,00E+04 |  | 0,00E+00 | 11,9 | 44 | 43 | 49 | 83 | 21,30 | 160 | 3 | 320 | < 2 |  | 1 | 4 | 6 |
| 12 | m | 41 |  |  | 0,00E+00 |  | 3,70E+04 | 6,5 | 27 | 32 | 86 | 23 | 13,70 | 80 |  | 0 |  |  |  |  |  |
| 13 | m | 34 | INF 2010 |  | 0,00E+00 |  | 0,00E+00 | 6,3 | 66 | 198 | 74 | 157 | 14,0 | 160 | 1 | 80 | < 2 |  |  |  |  |
| 14 | f | 50 |  |  | 0,00E+00 |  | 3,78E+02 | 5,4 | 13 | 19 | 119 | 12 | 14,1 | 160 | 1 | 80 | 3 | 0 |  |  |  |
| 15 | f | 38 |  |  | 0,00E+00 |  | 6,00E+01 | 3,5 | 18 | 20 | 49 | 36 | 14,6 | 160 | 1 | 80 | < 2 |  |  |  |  |
| 16 | m | 42 | INF 2010 | 1 | 1,70E+05 |  | 7,00E+01 | 7,8 | 93 | 180 | 68 | 58 | 14,9 | 160 | 1 | 160 | < 2 | 0 | 1 | 1 | 9 |
| 17 | m | 47 |  |  | 0,00E+00 |  | 0,00E+00 | 6 | 29 | 48 | 71 | 41 | 15,4 | 80 |  | 0 | 2,4 |  |  |  |  |
| 18 | m | 41 |  |  | 0,00E+00 |  | 9,10E+03 | 6,6 | 31 | 51 | 81 | 40 | 15,6 | 320 | 1 | 0 | < 2 | 0 |  |  |  |
| 19 | f | 58 |  |  | 1,20E+05 |  | 0,00E+00 | 3,3 | 46 | 61 | 78 | 35 | 16,8 | 80 |  | 0 | < 2 |  |  |  |  |
| 20 | f | 67 |  |  | 0,00E+00 |  | 0,00E+00 | 14 | 67 | 74 | 72 | 122 | 16,9 | 320 | 1 | 160 | < 2 |  |  |  |  |
| 21 | m | 55 | INF 2015 | 1 | 2,70E+05 |  | 6,00E+01 | 14,3 | 57 | 143 | 68 | 106 | 17,3 | 0 |  | 0 | < 2 |  |  |  | - |
| 22 | f | 27 |  |  | 6,70E+04 |  | 3,80E+02 | 18,5 | 64 | 80 | 143 | 158 | 17,4 | 160 | 1 | 0 | < 2 |  |  |  |  |
| 23 | f | 40 | INF 2011 | 1 | 3,20E+06 |  | 2,00E+01 | 29,1 | 58 | 58 | 72 | 58 | 18,6 | 320 | 1 | 40 | < 2 |  | 1 | 2 | 5 |
| 24 | m | 38 | Bulevirtide |  | 1,40E+03 |  | 2,00E+02 | 6,9 | 35 | 67 | 54 | 68 | 18,6 |  |  |  |  |  |  |  |  |
| 25 | f | 65 | INF 2015 | 1 | 3,20E+04 |  | 2,00E+01 | 17,3 | 101 | 135 | 82 | 44 | 18,8 | 320 | 1 | 0 | 9,3 | 0 | 1 | 4 | ? |
| 26 | m | 41 | INF 2011 | 1 | 0,00E+00 | D | 0,00E+00 | 7,5 | 124 | 281 | 57 | 47 | 19,5 | 640 | 1 | 0 | < 2 |  | 1 | 2 | 6 |
| 27 | m | 40 | INF | 5 | 5,40E+02 | D | 1,10E+04 | 7,6 | 35 | 42 | 79 | 24 | 20,0 | 160 | 1 | 0 | < 2 |  |  |  |  |
| 28 | f | 42 | INF 2016 |  | 2,20E+04 |  | 1,10E+02 | 7,8 | 93 | 146 | 66 | 30 | 22,3 | 80 |  | 0 | < 2 |  |  |  |  |
| 29 | m | 37 | INF 2010 | 1 | 4,50E+04 |  | 0,00E+00 | 8,4 | 94 | 141 | 145 | 355 | 22,4 | 160 | 1 | 80 | < 2 | 0 | 1 | 2 | 7 |
| 30 | m | 35 | INF |  | 4,80E+04 |  | 0,00E+00 | 31,1 | 88 | 118 | 122 | 354 | 22,5 | 320 | 1 | 640 | < 2 |  | 1 | 4 | 6 |
| 31 | m | 59 | INF 2011 |  | 0,00E+00 |  | 0,00E+00 | 14 | 52 | 32 | 74 | 304 | 24,40 | 160 | 1 | 40 | < 2 |  |  |  |  |
| 32 | m | 53 | INF | 1 | 1,20E+03 |  | 1,50E+02 |  | 99 | 123 | 220 | 80 | 25,00 | 640 | 1 | 0 | 2,8 | 0 |  |  |  |
| 33 | m | 52 | INF 2014 | 5 | 1,60E+05 |  | 1,20E+03 | 17,8 | 125 | 149 | 137 | 391 | 25,1 | 160 | 1 | 160 | < 2 |  | 1 | 2 | 7 |
| 34 | f | 33 |  |  | 1,60E+03 |  | 1,10E+01 | 14 | 288 | 114 | 307 | 159 | 25,9 | 80 |  | 320 | < 2 |  |  |  |  |
| 35 | m | 33 | INF 2014 | 1 | 5,20E+05 |  | 3,20E+02 | 13,9 | 51 | 94 | 115 | 30 | 27,3 | 80 |  | 0 | < 2 |  |  |  | - |
| 36 | m | 46 |  |  | 1,22E+04 |  | 1,69E+06 |  | 144 | 110 | 144 | 157 | 29,8 | 1280 | 1 | 0 | 3,9 |  |  |  |  |
| 37 | m | 33 |  |  | 4,00E+02 |  | 0,00E+00 | 14 | 99 | 63 | 135 | 35 | 36,5 | 1280 | 1 | 0 | 8,6 |  | 1 | 4 | 10 |
| 38 | f | 48 |  |  | 0,00E+00 |  | 5,64E+02 | 6,3 | 21 | 19 | 107 | 16 |  | 160 | 1 | 0 | < 2 | 0 |  |  |  |
| 39 | f | 58 |  |  | 7,70E+02 |  | 2,65E+02 |  | 30 | 38 | 57 | 28 |  | 640 | 1 | 0 | < 2 | 1 |  |  |  |
| 40 | m | 28 |  |  | 0,00E+00 |  | 1,38E+03 | 4,9 | 19 | 29 | 132 | 20 |  | 80 |  | 80 | < 2 |  |  |  |  |
| 41 | f | 32 |  |  | 0,00E+00 |  | 0,00E+00 | 12,4 | 174 | 243 | 158 | 193 | 14,7 | 160 | 1 | 0 | 2,3 |  |  |  |  |
| 42 | f | 27 | IFN |  | 1,50E+04 |  | 9,00E+01 |  | 45 | 70 |  | 102 |  |  |  |  |  |  | 1 | 4 |  |
