# Supplementary material for: Non-organ-specific autoantibodies with unspecific patterns are a frequent para-infectious feature of chronic hepatitis D
Source: Front Med (Lausanne). 2023 Jun 14;10:1169096. doi: 10.3389/fmed.2023.1169096 (PMC10300640; doi:10.3389/fmed.2023.1169096)
Supplement: Supplementary file 3 [file Table_3.DOCX]

| **No** | **Sex** | **Age** | **Therapy** | **HBV viral load (IU/ml)** | **liver stiffness (kPa)** | **ASAT** | **ALAT** | **AP** | **GGT** | **IgG** | **ANA Titer** | **ANA Pattern** | **SMA** | **SLA** | **cryoglobulins** | **Biopsy** | **Fibrosis** | **mHAI** |
| --- | --- | --- | --- | --- | --- | --- | --- | --- | --- | --- | --- | --- | --- | --- | --- | --- | --- | --- |
| 1 | f | 58 |  | 4,09E+03 | 6,1 | 28 | 44 | 47 | 41 | 12,60 | 0 |  | 0 | < 2 |  |  |  |  |
| 2 | f | 47 |  | 1,00E+03 | 4,4 | 21 | 14 | 54 | 15 | 9,70 | 80 |  | 80 | < 2 |  |  |  |  |
| 3 | f | 49 |  | 2,00E+04 |  | 14 | 17 | 47 | 45 | 14,20 | 320 | 1 | 0 | < 2 |  |  |  |  |
| 4 | f | 35 | Tenofovir | 0,00E+00 | 4,6 | 18 | 8 | 62 | 14 | 16,40 | 80 | 2 | 0 | 2,8 |  | 1 | 0 | 6 |
| 5 | m | 44 | Entecavir | 0,00E+00 | 5,4 | 11 | 9 | 15 | 88 | 12,30 | 320 | 2 | 0 | < 2 |  |  |  |  |
| 6 | m | 40 | IFN 2010 | 1,30E+02 | 10,1 | 19 | 32 | 56 | 14 | 10,20 | 160 | 2 | 160 | < 2 |  |  |  |  |
| 7 | m | 33 | IFN 2003, Tenofovir | 7,00E+01 | 5,2 | 18 | 32 | 82 | 17 | 12,40 | 0 |  | 0 | < 2 |  |  |  |  |
| 8 | f | 47 |  | 2,10E+04 | 3,4 | 19 | 21 | 71 | 18 | 13,80 | 160 | 2 | 0 | < 2 | 0 |  |  |  |
| 9 | m | 41 |  | 2,00E+04 | 6,6 | 22 | 40 | 49 | 37 | 14,50 | 160 | 2 | 0 | < 2 |  |  |  |  |
| 10 | m | 27 |  | 8,60E+02 | 5 | 39 | 111 | 67 | 24 | 10,00 | 160 | 3 | 0 | < 2 |  |  |  |  |
| 11 | f | 36 |  | 1,30E+03 | 5,9 | 19 | 20 | 58 | 20 | 10,60 | 0 |  | 0 | < 2 |  |  |  |  |
| 12 | m | 57 |  | 1,30E+02 |  | 17 | 27 | 60 | 42 | 7,50 | 640 | 4 | 0 | < 2 |  |  |  |  |
| 13 | m | 57 |  | 9,00E+01 | 5,4 | 19 | 31 | 92 | 72 | 18,40 | 160 | 2 | 0 | < 2 |  |  |  |  |
| 14 | m | 36 | Tenofovir | 0,00E+00 | 9,6 | 31 | 39 | 72 | 34 | 12,70 | 80 |  | 0 |  |  |  |  |  |
| 15 | f | 46 |  | 3,70E+03 |  | 22 | 41 | 50 | 15 | 13,60 | 160 | 3 | 0 | < 2 |  |  |  |  |
| 16 | m | 44 |  | 1,70E+03 |  | 22 | 15 | 64 | 40 | 10,40 | 0 |  | 0 | < 2 |  |  |  |  |
| 17 | f | 48 |  | 9,00E+01 |  | 21 | 21 | 126 | 18 | 12,90 | 80 |  | 0 | < 2 |  |  |  |  |
| 18 | m | 33 |  | 0,00E+00 |  | 30 | 46 | 75 | 28 | 8,26 | 0 |  | 0 | < 2 |  |  |  |  |
| 19 | f | 54 | Entecavir | 0,00E+00 |  | 22 | 24 | 74 | 18 | 13,40 | 0 |  | 0 | < 2 |  |  |  |  |
| 20 | m | 61 |  | 1,10E+02 |  | 17 | 30 | 31 | 58 | 14,10 | 0 |  | 0 | < 2 |  |  |  |  |
| 21 | m | 28 |  | 5,00E+03 |  | 36 | 56 | 76 | 69 | 10,19 | 80 |  | 0 | < 2 |  |  |  |  |
| 22 | f | 42 |  | 6,00E+05 | 6,9 | 16 | 17 | 60 | 22 | 9,36 | 80 |  | 0 | < 2 |  |  |  |  |
| 23 | f | 37 |  | 4,50E+02 | 6,1 | 15 | 19 | 58 | 23 | 11,90 | 0 |  | 0 | 2 |  |  |  |  |
| 24 | m | 33 |  | 3,00E+09 |  | 49 | 107 | 54 | 27 | 13,70 | 0 |  | 0 | < 2 |  | 1 | 0 |  |
| 25 | m | 47 |  | 7,60E+02 | 4 | 44 | 94 | 68 | 30 | 10,50 | 160 | 2 | 0 | < 2 |  |  |  |  |
| 26 | f | 20 |  | 2,00E+10 | 3,8 | 13 | 20 | 96 | 25 | 17,20 | 640 | 1 | 0 | < 2 |  |  |  |  |
| 27 | m | 39 |  | 2,00E+04 | 6,3 | 38 | 61 | 51 | 58 | 13,10 | 80 |  | 80 | < 2 |  |  |  |  |
| 28 | m | 52 |  | 3,70E+02 | 3,2 | 18 | 19 | 49 | 25 | 10,20 | 320 | 2 | 0 | - |  |  |  |  |
| 29 | f | 19 |  | 1,20E+09 | 5 | 27 | 59 | 60 | 14 | 10,30 | 0 |  | 0 | < 2 |  |  |  |  |
| 30 | f | 38 |  | 7,50E+04 |  | 9 | 17 | 35 | 10 | 14,80 | 0 |  | 0 | < 2 |  |  |  |  |
| 31 | m | 30 |  | 1,00E+02 | 6,1 | 20 | 19 | 66 | 31 | 12,90 |  |  |  |  |  |  |  |  |
| 32 | f | 34 |  | 3,00E+05 |  | 23 | 43 | 80 | 22 | 12,50 | 160 | 2 | 80 | < 2 |  |  |  |  |
| 33 | f | 30 |  | 5,70E+02 | 5,6 | 25 | 40 | 82 | 19 | 14,70 | 80 |  | 0 | < 2 |  |  |  |  |
| 34 | f | 48 | Tenofovir | 6,00E+01 | 6,8 | 29 | 25 | 86 | 16 | 11,20 | 0 |  | 0 | 2,6 |  |  |  |  |
| 35 | m | 48 |  | 1,00E+07 | 16,8 | 245 | 363 | 71 | 67 | 24,70 | 0 |  | 0 | 3,12 | 1 |  |  |  |
| 36 | m | 28 |  | 2,60E+04 | 4,3 | 26 | 50 | 80 | 47 | 14,00 | 160 | 2 | 0 | 2,8 |  |  |  |  |
| 37 | m | 19 |  | 8,10E+08 | 4,3 | 45 | 122 | 83 | 41 | 15,40 | 160 | 2 | 0 | < 2 |  | 1 | 0 | 3 |
| 38 | f | 36 | Tenofovir | 1,18E+03 |  | 20 | 23 | 67 | 12 |  | 0 |  | 0 |  | 0 |  |  |  |
| 39 | m | 35 |  | 2,40E+04 | 3,6 | 40 | 49 | 106 | 27 | 19,10 | 80 | 2 | 0 | < 2 |  |  |  |  |
| 40 | f | 61 |  | 4,40E+02 |  | 25 | 42 | 73 | 37 | 14,40 | 80 |  | 0 | < 2 |  |  |  |  |
| 41 | f | 66 |  | 1,30E+03 |  | 32 | 45 | 93 | 78 | 7,93 | 80 |  | 0 | < 2 |  |  |  |  |
| 42 | f | 31 |  | 1,80E+04 | 4,9 | 18 | 19 | 40 | 17 | 6,74 | 320 | 2 | 0 | < 2 |  |  |  |  |
| 43 | m | 29 |  | 2,70E+08 |  | 49 | 127 | 73 | 38 | 9,22 | 80 |  | 0 | < 2 |  |  |  |  |
| 44 | f | 35 |  | 1,40E+03 | 4,9 | 17 | 14 | 70 | 16 | 10,2 | 160 | 2 | 80 | < 2 |  |  |  |  |
| 45 | m | 57 |  | 9,40E+03 |  | 32 | 31 | 71 | 24 | 7,8 | 160 | 2 | 0 | - |  |  |  |  |
| 46 | f | 42 |  | 2,30E+03 | 15,9 | 18 | 9 | 75 | 16 | 14 | 320 | 1 | 0 | < 2 |  |  |  |  |
| 47 | m | 28 |  | 9,00E+02 | 5,9 | 26 | 37 | 68 | 29 | 21 | 320 | 2 | 0 | < 2 |  |  |  |  |
| 48 | m | 33 |  | 3,70E+05 | 3,9 | 60 | 63 | 48 | 123 |  | 320 | 2 | 0 | < 2 |  |  |  |  |
| 49 | m | 41 |  | 1,30E+04 | 4,8 | 23 | 46 | 60 | 32 |  | 160 | 2 | 160 | - |  |  |  |  |
| 50 | f | 26 | Tenofovir | 4,56E+02 | 4,3 | 19 | 23 | 78 | 21 |  | 160 | 2 | 80 | < 2 |  |  |  |  |
| 51 | m | 19 |  | 8,29E+02 |  | 320 | 120 | 86 | 22 | 13,3 | 320 | 2 | 0 | < 2 |  |  |  |  |
| 52 | f | 52 |  | 4,10E+03 | 4,2 | 14 | 9 | 87 | 20 |  | 320 | 2 | 0 | < 2 |  |  |  |  |
| 53 | m | 42 |  | 1,30E+04 | 14,3 | 17 | 19 | 89 | 55 | 12,1 | 640 | 3 | 0 | < 2 |  |  |  |  |
| 54 | f | 19 |  | 1,50E+02 | 4,3 | 33 | 57 | 53 | 30 | 14 | 320 | 2 | 0 | < 2 |  |  |  |  |
| 55 | m | 34 |  | 1,30E+03 | 5,8 | 17 | 12 | 53 | 17 | 10,7 | 160 | 2 | 0 | < 2 |  |  |  |  |
| 56 | f | 41 | Lamivudin | 0,00E+00 |  | 19 | 19 | 53 | 19 | 7,80 | 0 |  | 0 | < 2 |  |  |  |  |
| 57 | m | 56 |  | 3,00E+06 | 21,3 | 62 | 95 | 69 | 122 | 19,30 | 0 |  | 40 | < 2 |  |  |  |  |
| 58 | f | 31 |  | 1,00E+02 |  | 27 | 39 | 49 | 85 | 13,10 | 0 |  | 80 | < 2 |  |  |  |  |
| 59 | f | 33 |  | 2,50E+03 |  | 20 | 24 | 88 | 33 | 12,90 | 0 |  | 0 | 2,6 |  |  |  |  |
| 60 | f | 31 |  | 1,00E+04 | 4,1 | 14 | 19 | 65 | 17 |  | 0 |  | 80 |  |  |  |  |  |
| 61 | f | 30 |  | 3,00E+03 |  | 9 | 15 | 63 | 13 | 16,00 | 80 |  | 0 | < 2 |  |  |  |  |
| 62 | f | 62 | Entecavir | 0,00E+00 | 6,6 | 14 | 24 | 66 | 17 |  | 0 |  | 0 |  |  |  |  |  |
| 63 | f | 44 |  | 0,00E+00 |  | 21 | 13 | 30 | 13 | 8,42 | 320 | 2 | 0 | 6,5 |  |  |  |  |
| 64 | f | 64 | Entecavir | 3,00E+08 |  | 1376 | 942 | 170 | 77 | 8,44 | 80 |  | 0 | 4,3 |  |  |  |  |
| 65 | f | 29 |  | 2,00E+04 |  | 17 | 19 | 33 | 29 | 12,40 | 80 |  | 0 | 3,1 |  |  |  |  |
| 66 | f | 47 |  | 500 000 000 |  | 164 | 290 | 38 | 30 | 11,66 | 80 |  | 40 | 3,61 |  |  |  |  |
| 67 | f | 29 |  | 4,00E+01 |  | 14 | 20 | 66 | 17 | 15,50 | 0 |  | 0 | < 2 | 0 |  |  |  |
| 68 | f | 36 |  | 50 000 |  | 30 | 40 | 62 | 21 |  | 80 |  | 40 |  |  |  |  |  |
| 69 | f | 20 |  | 1,00E+03 |  | 21 | 17 | 62 | 20 |  | 160 |  | 80 | 3,8 |  |  |  |  |
| 70 | f | 30 |  | 90 000 |  | 27 | 18 | 49 | 14 | 16,88 | 80 |  | 0 | 2,6 |  |  |  |  |
