# Supplementary material for: Non-organ-specific autoantibodies with unspecific patterns are a frequent para-infectious feature of chronic hepatitis D
Source: Front Med (Lausanne). 2023 Jun 14;10:1169096. doi: 10.3389/fmed.2023.1169096 (PMC10300640; doi:10.3389/fmed.2023.1169096)
Supplement: Supplementary file 4 [file Table_4.DOCX]

|  | HDV PCR + | HDV PCR – | CHB |
| --- | --- | --- | --- |
| n= | 20 | 19 | 61 |
| IgG (U/l) | 20.7 (17.3-25.1)** | 13.7 (11.7-15.5) | 12.7 (10.2-14.3) |
| n= | 20 | 19 | 69 |
| ANA titer > 1:80 | 13 (65%) | 13 (68%) | 30 (43%)** |
| ANA titer ≥ 1:320 | 7 (35%) | 4 (21%) | 14 (20%) |
| SMA titer >1:80 | 5 (25%) | 1 (5%) | 2 (3%)* |
| SMA titer ≥ 1:320 | 3 (15%) | 0 (0%) | 0 (0%) |
| n= | 21 | 19 | 70 |
| ASAT (U/l) | 64 (44-99)** | 26 (20-34) | 22 (18-31) |
| ALAT (U/l) | 95 (62-138)** | 32 (20-48) | 29 (19-47) |

Supplementary Table 4: IgG levels, frequency of ANA titers, SMA titers, and transaminase levels in patients with HDV PCR+, HDV PCR-, and CHB. Values are medians with an interquartile range (IQR). Significance was tested with the Chi-square test for autoantibody titers and with Mann- Whitney- U test for IgG and transaminase levels: HDV PCR+ compared to HDV PCR- (*p > 0.05, **p > 0.01).
